# Future Trends in Synthetic Biology—A Report

**DOI:** 10.3389/fbioe.2019.00175

**Published:** 2019-08-07

**Authors:** Meriem El Karoui, Monica Hoyos-Flight, Liz Fletcher

**Affiliations:** ^1^SynthSys-Centre for Synthetic and Systems Biology, School of Biological Sciences, University of Edinburgh, Edinburgh, United Kingdom; ^2^Innogen Institute, School of Social and Political Sciences, University of Edinburgh, Edinburgh, United Kingdom

**Keywords:** synthetic biology, biosystem, future trends and developments, biodesign automation, responsible research and innovation (RRI)

## Abstract

Leading researchers working on synthetic biology and its applications gathered at the University of Edinburgh in May 2018 to discuss the latest challenges and opportunities in the field. In addition to the potential socio-economic benefits of synthetic biology, they also examined the ethics and security risks arising from the development of these technologies. Speakers from industry, academia and not-for-profit organizations presented their vision for the future of the field and provided guidance to funding and regulatory bodies to ensure that synthetic biology research is carried out responsibly and can realize its full potential. This report aims to capture the collective views and recommendations that emerged from the discussions that took place. The meeting was held under the Chatham House Rule (i.e., a private invite-only meeting where comments can be freely used but not attributed) to promote open discussion; the findings and quotes included in the report are therefore not attributed to individuals. The goal of the meeting was to identify research priorities and bottlenecks. It also provided the opportunity to discuss how best to manage risk and earn public acceptance of this emerging and disruptive technology.

## Introduction

**Figure 1:**
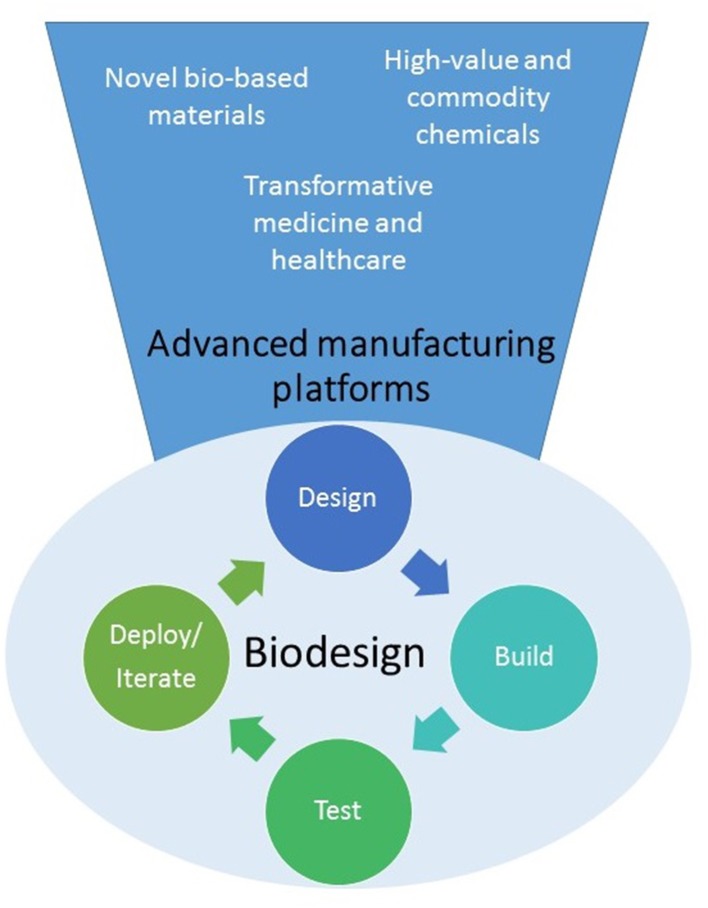
Synthetic biology is developing into a biodesign platform where it will be possible to apply the “design-build-test-iterate (or deploy)” to predictably create cells or organisms able to produce a wide variety of novel molecules, materials or even cells for multiple applications.

Synthetic Biology offers innovative approaches for engineering new biological systems or re-designing existing ones for useful purposes (see Figure 1). It has been described as a disruptive technology at the heart of the so-called Bioeconomy, capable of delivering new solutions to global healthcare, agriculture, manufacturing, and environmental challenges (Cameron et al., 2014; Bueso and Tangney, 2017; French, 2019). However, despite successes in the production of some high value chemicals and drugs, there is a perception that synthetic biology is still not yet delivering on its promise.

Moreover, there are some concerns from governments that synthetic biology expands the pool of agents of concern, which increases the need to develop detection, identification and monitoring systems, and proactively build countermeasures against chemical and biological threats (Wang and Zhang, 2019). The participation of representatives from various government organizations at this meeting is testament to their commitment to maintaining an active dialogue with the synthetic biology community. In this way, they aim to keep abreast of the changing nature of threats and provide the best advice to government about investment in science and technology and the introduction or amendment of regulatory processes.

The cost of DNA sequencing and synthesis have decreased dramatically (Carlson, 2014; Kosuri and Church, 2014) and we have access to more genetic information and more powerful genetic engineering capabilities than ever before. Critical investments in infrastructure are bearing fruit and, as is described below, synthetic biology is increasingly becoming, at least part of, the solution to many of our present and future needs in medicine, food and energy production, remediation, manufacturing, and national security. So what is the potential of synthetic biology and what challenges does it still face to realize this?

## Advances in Synthetic Biology: The State of Play

### Small Molecules: Production on Demand a Reality

Despite the lack of predictability in biology, and current technical constraints that limit data collection and analyses, we can now produce small molecules on demand using synthetic biology approaches.

Probably the most impressive examples come from the Foundry at the Broad Institute of MIT and Harvard. When the Defense Advanced Research Projects Agency (DARPA) put the MIT-Broad Institute Foundry's design capabilities to the test, its researchers were able to deliver 6 out of 10 molecules of interest to the US Department of Defense in 90 days. This “pressure test” confirms the potential of synthetic biology to address shortages of key compounds quickly (Casini et al., 2018).

Indeed, many labs can now design and construct relatively complex gene networks capable of generating a wide variety of “designer” molecules in a range of host cells; however, this is often a slow iterative process of trial and error.

As yet, very few small molecules in medicine are manufactured using a synthetic biology process; it remains very difficult to engineer microbes to carry out processes that Nature did not intend. This is to be expected: the performance of microbes is “good enough” from an evolutionary perspective. Microbes evolved to address the specific needs and challenges of their natural environments not those of industrial fermenters and bioreactors. Gene Transfer from one system to another may sound easy but in practice is hard work and rarely generates sufficient reward (i.e., increased yield) to justify the investment made. The application of automation and artificial intelligence (e.g., in designing and building plasmids) may help to reduce the time and cost—and improve return on investment—in the future (Zhang et al., 2018).

“*Scale up is product specific – we need more synthetic biology in the production process”*

Plants make alternative production platforms. Improvements in mining plant genomes and the development of effective transient expression systems have enabled large-scale production of, for example, vaccines in tobacco plants in just a few weeks (Dirisala et al., 2017; Emmanuel et al., 2018). Directing the production of synthetic biological materials to plant chloroplasts also shows promise (Boehm and Bock, 2019).

The photosynthetic reducing power generated in plant chloroplasts can be harnessed for the light-driven synthesis of bioactive molecules such as dhurrin, which protects plants against insects (Gnanasekaran et al., 2016).

However, underlying all these platforms is a knowledge gap in our understanding in how nature works. This makes it very hard to apply the design/build/test/learn cycles used in conventional engineering to the production of synthetic biological materials whatever the production platform (yeast, bacteria, plants, or human cells) if the platform itself is not well-understood (Sauro et al., 2006).

What we need now are instruments able to measure and characterize outputs, assisted by progress in robotics and automation, and the application of machine learning approaches to analyse the data generated. This will help us to generate more robust models of biological systems, so we can improve experimental design for future engineering strategies.

“*We can do ‘build’. ‘Test’ is the challenge when we want to learn from the iterative design process”*

### Healthcare: Reimagining Medicine

Synthetic biology is driving significant advances in biomedicine, which will lead to transformational improvements in healthcare. Already, patients are benefiting from so-called CAR (for chimeric antigen receptor) technology, which engineers the immune cells (T-cells) of the patient to recognize and attack cancer cells (June et al., 2018).

Genetically engineered viruses are being used to correct defective genes in patients with inherited diseases such as Severe Combined Immune Deficiency (SCID) or epidermolysis bullosa (Dunbar et al., 2018).

The ability to reprogramme somatic cells from patients into induced pluripotent stem cells is furthering our understanding of their disease, reducing the use of animals in research, and paving the way for the development of personalized medicines and cell therapies. In principle at least, we could engineer a patient's own cells to multiply, differentiate into different cell types and even self-assemble into new tissues, or even organs, to repair those damaged through disease or injury (Davies and Cachat, 2016; Satoshi et al., 2018).

Work on new vectors that are able to deliver large genetic loads to target tissues is helping to produce more efficient therapeutics and vaccines that will have fewer side effects and a smaller risk of resistance. Furthermore, optimizing antibody or vaccine production, or example, so that they are in an edible format (e.g., plant based), could greatly reduce the cost and increase the speed of vaccine production in an epidemic.

“*We have the tools but need the creativity to make stuff that can't be made without synthetic biology”*

In the next few years, genetically engineering pigs to be virus resistant and have human-like immune profiles could make xenotransplantation a clinical reality (Burkard et al., 2018). Engineering the microbiome is expected to lead to the development of synthetic probiotics (Dou and Bennett, 2018).

The synthetic biology initiative known as Human Genome Project-write (HGP-write) has set its sights even higher, rallying scientists to build entire human chromosomes (Boeke et al., 2016). Concerns have been raised about the ethics of creating “synthetic humans” and indeed the scientific and commercial value of such a project. More recently, HGP-write champions have proposed a more focused project to build a virus-resistant chromosome, making at least 400,000 changes to the human genome to remove DNA sequences that viruses use to hijack cells and replicate (Dolgin, 2018).

One of the many exciting opportunities that synthetic biology offers medicine is in the production of theranostic cell lines that can sense a disease state and produce an appropriate therapeutic response (Teixeira and Fussenegger, 2019). Several obstacles need to be overcome to achieve this goal: first, to expand the range of molecules that can be recognized by cellular “sensors” as inputs; and second, to better understand the genetic control factors that regulate gene expression in space and time so we can engineer better activator systems.

“*At present we need pills because we can't swallow a chemistry kit”*

Metabolomics is shedding light on many disease biomarkers. Because some biomarkers are shared between seemingly unrelated diseases, an accurate diagnosis will require the detection of multiple markers to provide a more unique “disease fingerprint.” Work in whole cell and cell free systems to develop sensors of multiple disease-specific biomarkers could assist in earlier detection of disease and prognostic monitoring.

To expand the range of biologically detectable molecules, it is possible to design metabolic pathways that transform currently undetectable molecules of interest (e.g., hippuric acid, the prostate cancer biomarker) into molecules for which sensors already exist (in this case benzoic acid) (Libis et al., 2016).

Cybergenetics is an emerging field that is developing experimental tools for the computer control of cellular processes at the gene level in real time. Cybergenetic control can be achieved by interfacing living cells with a digital computer that switches on or off the embedded “genetic switch” using light (optogenetics) or chemicals (Gabriele et al., 2018; Maysam et al., 2019). Such systems could help to maintain cellular homeostasis by monitoring the state of the body and triggering an appropriate response upon the detection of dysregulation; for example, they could trigger the release of insulin when blood glucose levels rise as detected by a wireless diagnostic tool (Ye et al., 2011).

### Advanced Materials: Inspired by Nature, Improved by Synthetic Biology

Synthetic biology offers the opportunity to create responsive and multifunctional materials (Le Feuvre and Scrutton, 2018). The integration of biochemical components from living systems with inorganic components can lead to new materials that are able to sense the environment (or internal signals) and change their properties. These features could be particularly useful for improving protective clothing or building materials.

An issue when using microbes to produce composite materials is regulating the assembly of these materials to achieve specific desired properties. By understanding how microbes communicate with each other, it is possible to make them work better together and combine them with other production systems so that the properties of materials can be tailored for particular functions.

Interestingly, rather than modifying, or improving existing protein-based materials, an alternative approach involves using computational techniques to design completely novel proteins that self-assemble into predicted shapes (Ljubetič et al., 2017). Such “programmable” proteins open up even further opportunities for synthetic biology not only for materials science but also for medicine and chemistry.

“*The tools are there, we just don't know what we want to make”*

## Tackling the Challenges

Regardless of the research areas involved, there are some common challenges for the community to address.

### Design With the End in Mind

There was consensus that while the production of small molecules using synthetically engineered cells at the bench is becoming more tractable, these processes often do not translate well into mass production. Scalability needs to be incorporated into the initial design process by including features, for example, that reduce toxicity of the molecule to the production host or “chassis” and/or by introducing modifications that favor its extraction. Careful consideration of the right chassis could also greatly improve yields and significantly reduce cost of production.

“*Nature has developed its own ways to concentrate and solubilise chemicals…we are not learning from this”*

### Expanding the Host Repertoire

The number of microbes that are currently in use for the production of synthetic biological materials is only a tiny fraction of the total diversity that exists in Nature; only ten microbes are “domesticated” for industrial use. To identify the most appropriate chassis, it is worth turning to nature to identify species that have unique metabolic networks suited to host particular types of chemical reactions. For example, the soil bacterium *Pseudomonas putida*, which has adapted to harsh environmental conditions, is ideally suited to host redox-intensive reactions (Pablo and de Lorenzo, 2018). A treasure trove of, as yet, unexplored natural products, with novel and beneficial properties, exist in the plant kingdom. In addition, as noted above, plants are naturally excellent production hosts and it is now possible to “plug and play” combinations of plant pathways to generate novel molecules (Sainsbury and Lomonossoff, 2014; Evangelos and O'connor, 2016).

### Developing a Universal Production System

To circumvent the problem of the impact of different host chassis on synthetic gene circuits, researchers would benefit from a universal synthetic expression system that permits the testing of new constructs. This would aid in the identification of optimal production platforms and decrease the need for organism-specific technologies. Combining this technology with DNA editing techniques, such as CRISPR/Cas9, will make the establishment of host production platforms and the generation of complex biosynthetic products easier and faster. One articulation of this could be in cell-free formats, in which the essential cellular machinery is reconstituted *in vitro* and used as a manufacturing platform (Villarreal and Tan, 2017; Koch et al., 2018).

“*We need to reduce the burden of synthetic networks on endogenous circuits”*

### Move to Cell-Free Environments

Cell-free environments, interfaced with semiconductors, offer a powerful route for flexible and controllable production systems.

For example, nanoparticles made of semiconductor materials or quantum dots can be used to enhance enzyme activity in a cell free environment with a minimal set of ingredients. Multistep enzymatic pathways can be tethered to nanoparticle surfaces and, by avoiding the diffusion effect that takes place in cells, reaction rates can be increased 100-fold (Wang et al., 2017). This approach can be used to access non-natural materials and circumvents the potential issue of toxicity in cells (as well as the regulatory issues of genetically modified cells).

Research on silicon chips containing immobilized genes and cell lysates allows detailed examination of gene expression in space and time. When the compartments in which DNA-driven reactions take place are linked, with materials flowing into and diffusing between the compartments, it is possible to recreate oscillating protein expression patterns and protein gradients akin to those observed in cells (Karzbrun et al., 2014). Gene expression in these “artificial cells” can be controlled with electrodes that prevent the assembly of proteins by ribosomes. By standardizing outputs, this strategy is improving the predictability of engineered genetic circuits.

### Systems Modeling, Standards, and Metrology

Whatever the system being explored, a robust model of that biological system is needed if predictable modifications of genetic circuits are to be designed and implemented with any confidence. To create such models, large sets of data arising from measuring multiple parameters of cell behavior under different conditions are essential (Fletcher et al., 2016).

There is great expectation that improvements in high-throughput data measurement and collection systems will generate exactly the large data sets needed. These can be analyzed using artificial intelligence or a machine learning approach to optimize the design of synthetic biological products and move away from the inefficient trial and error process (Decoene et al., 2018).

Finally, agreement on standards of design, assembly, data transfer, data measurement and regulatory rules, as well as on the language that is used, will help to improve the interdisciplinary and international collaborations that are required to drive the field forward. This is challenging for a community with such diverse interests and perspectives, and where data sharing, curating and quality control is not common practice.

However, without some form of agreed standards, many of the products and processes of synthetic biology will not translate well to industrial settings dependent on reproducible processes and beholden to exacting regulatory requirements.

Typically, academic researchers are driven by a need to understand the complexity of nature (and publish their work in high impact journals). Standardization and scaling up production are important, but have less of an academic pull than discovering a new product. Access to funding, industrial partnerships, and academic recognition are examples of potential incentives for carrying out this type of research.

“*Standards restrict flexibility but enable interoperability”*

## Tackling Risk

Synthetic biology is an example of a dual-use technology: it promises numerous beneficial applications, but it can also cause harm. This has led to fears that it could, intentionally or unintentionally, harm humans or damage the environment. For example, there is huge value in our ability to engineer viruses to be more effective and specific shuttles for gene therapies of devastating inherited disorders; however, engineering viruses may also lead to the creation of even more deadly pathogens by those intent on harm.

“*Synthetic biology should be regarded as an extension of earlier developments and technologies”*

Some would argue that synthetic biology poses an existential risk and needs to be treated with extreme caution. However, many new technological advances across the decades have met similar concerns. The uncertainty and remote possibility of such risks could hamper the development of useful technology. Scientists, their host institutions and funding bodies should (and indeed already do) consider whether the research planned could be misused. Measures that reduce the likelihood of misuse and its consequences should be implemented and clearly communicated. The synthetic biology community needs to be aware of, and respond to, these challenges by engaging in horizon scanning exercises as well as open dialogue with regulatory bodies and the media.

“*Don't avoid risk – manage it”*

Being more open about risks, and how they are controlled, provides an opportunity to shift discourse toward the benefits of synthetic biology in addressing urgent global needs, such as the production of biofuels, food security and more effective medicines, and potentially improve public acceptance.

“*The questions should not be ‘what’s the next big thing for synthetic biology' but ‘where is the greatest unmet need’.”*

Despite the efforts by individual countries to establish synthetic biology research roadmaps, broader, international agreement on common standards (and red lines) across the field may help establish trust and to advance the best pre-competitive research into useful applications.

Meeting participants highlighted the importance of training in responsible research conduct and ethics. Given students' future role as science ambassadors and influencers, their training should not only convey skills and knowledge but also awareness and critical thinking about the prospects and potential for dual use of synthetic biology. All researchers must remain vigilant regardless of the many pressures and distractions of running a successful research lab; they may not have specialist training in identifying the risks of misuse but they are the people best placed to maintain informed oversight of risks.

One example of current synthetic biology research with potential dual use is gene drive technology, which can be used to propagate a particular suite of genes throughout a population. The benefits of using gene drive technology include the eradication of disease-carrying insect populations and the elimination of invading pest species but it has raised concerns about the unintended ecological impacts of reducing or eliminating a population (Callaway, 2018; Collins, 2018).

Similar release concerns surround research that is harnessing the ability of pathogens to target particular tissues in the body or particular chemicals in the environment, which could greatly aid efforts to deliver targeted therapies or clean-up contaminated sites. To date, such large-scale release for environmental bioremediation interventions has not been possible.

“*We need to mind the gap between R&D scale up and communications …. One bad blog can kill a commercial product”*

There was consensus that the need for regulation over this community remains important. Regulation needs to keep up to speed with the emerging technologies and should focus on the product rather than the process used to create it (Tait et al., 2017). Unsuitable regulatory frameworks (as well as unfavorable public perception) could discourage private sector investment in synthetic biology.

Open and balanced two-way communication between researchers, funders, companies and governments and the public will be vital. Consumers and activists may have no interest in the difference between making a chemical (e.g., a flavor or fragrance) synthetically and one made using genetically modified bacteria but they may instinctively distrust the latter.

## Conclusions and Recommendations

The short talks presented at this 2-day meeting suggest that synthetic biology is at the cusp of many major breakthroughs and that it is perhaps timely to re-define the meaning of “success” in synthetic biology.

There are many hurdles to overcome but the potential for synthetic biology to deliver solutions to many global challenges—improving healthcare, limiting environmental damage, and creating a wide variety of more sustainable processes—is great.

Meeting participants suggested that as a community they should support the measures listed below to help synthetic biology move beyond the proof-of-concept stage and to ensure that potential risks are minimized and dialog with the public can be optimized.

- Larger and longer investment in better big data management and processing (Artificial Intelligence and Machine Learning) systems, and in fundamental research on biosystems modeling, chassis development, and genome mining.
- Support standardization initiatives; while arguably not attractive academically, the community needs to agree and support efforts toward creating interoperability around biosystem modeling, and standards around DNA design (if not DNA bioparts). Without some degree of “standardization” the ability to pool data and models, essential for improving accuracy and reproducibility, will be challenging.
- The creation and funding (ideally internationally) of a “Grand Challenge,” such as the development of generic sensors or the creation of protein-based electronic components, could help focus the community toward a target goal.
- Improve the assessment, communication, and management of risk and harm among all audiences.
- Ensure that early career researchers are trained in responsible research conduct and ethics as well as being cognizant of existing rules and regulations around GM (regionally dependent) and the issue of misuse and harm.
- Coordinate the efforts of academia, government and industry through focused meetings that foster interdisciplinary collaborations around shared objectives.
- Improve platforms for knowledge sharing and recognize the value of failures.

The workshop highlighted just how much more we have to learn from Nature itself. Synthetic biology is giving us insights of the criteria and processes that underpin all living systems; in turn, we can take this insight and design and use it to build a “better biology.” However, we need to take the public with us on this journey, create meaningful and considered dialogue about the work we may do and the impact it might have on our world.

## Author's Note

The meeting was hosted by the UK Center for Mammalian Synthetic Biology (the Center), based at the University of Edinburgh. The Center is building expertise in cell engineering tool generation, whole-cell modeling, computer-assisted design and construction of DNA and high-throughput phenotyping to enable synthetic biology for medicine and healthcare. The Centre's research will not only advance basic understanding of mammalian biology and pathology but also generate products and services for near-term commercial exploitation by the pharmaceutical and drug testing industries, such as diagnostics, novel therapeutics, protein-based drugs, and regenerative medicines. The Center is funded through the Research Council's UK “Synthetic Biology for Growth” programme and by the Biological and Biotechnology Sciences Research Council (BBSRC), the Engineering and Physical Sciences Research Council and the Medical Research Council.

## Author Contributions

This is a meeting report and was drafted by MH-F with support from ME and LF.

## Conflict of Interest Statement

The authors declare that the research was conducted in the absence of any commercial or financial relationships that could be construed as a potential conflict of interest.
